# 胸腺肿瘤专题前言

**DOI:** 10.3779/j.issn.1009-3419.2016.07.01

**Published:** 2016-07-20

**Authors:** 文涛 方, 克能 陈

**Affiliations:** 1 200030 上海，上海交通大学附属上海胸科医院 Shanghai Jiaotong University Affiliated Shanghai Chest Hospital, Shanghai 200030, China; 2 100142 北京，北京大学附肿瘤医院胸外科 Department of Thoracic Surgery, Tumor Hospital Affiliated to Peking University, 100142 Beijing, China

胸腺肿瘤是比较少见的胸部实体肿瘤，同时也是临床上最常见的前纵隔肿瘤。但自然预后及治疗后的远期生存却均好于诸如肺癌、食管癌等其他胸部恶性肿瘤。由于发病率低，现行的临床病理分期、组织学分型及诊疗指南都是基于专家意见，而缺乏有力的循证医学证据来指导胸腺肿瘤的规范化临床诊治。即使有些研究也大多为单中心回顾性病例报道和观察性研究，更无任何前瞻性随机对照的临床试验结果。

解决这种少见肿瘤诊疗困境的唯一出路在于多中心多学科的合作。在这方面西方和日本同行做出了很好的榜样。自2009年国际胸腺肿瘤研究协会（International Thymic Malignancy Interest Group-ITMIG）成立以来，各地区的协作组织如欧洲胸外科医师协会胸腺工作组（European Society of Thoracic Surgeons-ESTS Thymic Working Group）、日本胸腺研究会（Japanese Association for Research of the Thymus）、韩国胸腺研究会（Karean Association for Research of the Thymus）等渐次成立，对胸腺肿瘤研究的区域性合作起到了重要的推动作用，其中也包括中国胸腺肿瘤研究协作组（Chinese Allinance for Research in Thymomas, ChART）。正是基于这些国际性和地区性的合作，对胸腺肿瘤的认识在近年有了较大的进步，首先是建立了全球性回顾性数据库，其中中国ChART的10家成员单位贡献了近2, 000例病例用于分析，基于这些大样本数据的分析，ITMIG发表了胸腺肿瘤的一系列指导性文献，包括纵隔解剖分区、组织学分类等共识，并提出了国际抗癌联盟（Union for International Cancer Control, UICC）首个胸腺肿瘤临床病理分期。

ChART自成立以来在短短3年多时间里联合全国20家医院共同建立了国内首个胸腺肿瘤回顾性数据库，并据此进行了多项大病例组回顾性分析，以英文发表于《*Jounal of Thoracic Disease*》2016，8（4），受到了国际学者的关注。为了让国内广的医务工作者大尤其是基层胸腺肿瘤从医人员更好的了解这些研究结果用以指导临床实践，ChART联合《*Jounal of Thoracic Disease*》杂志及《中国肺癌杂志》，在得到了版权转让权的前提下，将这批文献以中文形式再次在《中国肺癌杂志》刊发。这组文献涵盖术前诊断、临床分期和组织学分型、手术方式、术前诱导治疗和术后辅助治疗等各个方面，并在此基础上提出中国医师协会胸外科分会胸腺肿瘤诊治共识，希望对今后的临床诊疗和进一步的研究提供有益的帮助。

胸腺肿瘤的诊疗仍有许多问题亟待解决, 鉴于胸腺肿瘤的少见性且相对惰性，在临床研究中应继续加强协作，以更深入地认知这一疾病。与ITMIG全球数据库项目一样，ChART回顾性数据库分析也为罕见肿瘤（如胸腺疾病）的研究树立了榜样，并且为将来开展多中心前瞻性临床研究搭建了良好的平台。当前亟需加强不同医院、区域、国家之间的协作，争取组织大规模的临床研究来解决现有的问题，为进一步改进临床实践铺平道路。

